# Vaccines to prevent COVID-19: a protocol for a living systematic review with network meta-analysis including individual patient data (The LIVING VACCINE Project)

**DOI:** 10.1186/s13643-020-01516-1

**Published:** 2020-11-20

**Authors:** Steven Kwasi Korang, Sophie Juul, Emil Eik Nielsen, Joshua Feinberg, Faiza Siddiqui, Giok Ong, Sarah Klingenberg, Areti Angeliki Veroniki, Fanlong Bu, Lehana Thabane, Allan Randrup Thomsen, Janus C. Jakobsen, Christian Gluud

**Affiliations:** 1 Copenhagen Trial Unit, Centre for Clinical Intervention Research, Department 7812, Rigshospitalet, Copenhagen University Hospital, Blegdamsvej 9, 2100 Copenhagen, Denmark; 2 Systematic Review Initiative, NHS Blood and Transplant, John Radcliffe Hospital, Headley Way, Oxford, OX3 9BQ UK; 3 The Cochrane Hepato-Biliary Group, Copenhagen Trial Unit, Centre for Clinical Intervention Research, Department 7812, Rigshospitalet, Copenhagen University Hospital, Copenhagen, Denmark; 4 Department of Primary Education, School of Education, University of Ioannina, Ioannina, Greece; 5 Knowledge Translation Program, Li Ka Shing Knowledge Institute, St. Michael’s Hospital, Toronto, Ontario Canada; 6 Centre for Evidence-based Chinese Medicine, Beijing University of Chinese Medicine, Beijing, China; 7 Department of Health Research Methods, Evidence, and Impact, McMaster University, Hamilton, Ontario Canada; 8 Department of Immunology and Microbiology, University of Copenhagen, Copenhagen, Denmark; 9 Department of Regional Health Research, The Faculty of Health Sciences, University of Southern Denmark, Odense, Denmark

## Abstract

**Background:**

Severe acute respiratory syndrome coronavirus 2 (SARS-CoV-2) causes coronavirus disease 2019 (COVID-19) which has rapidly spread worldwide. Several human randomized clinical trials assessing potential vaccines are currently underway. There is an urgent need for a living systematic review that continuously assesses the beneficial and harmful effects of all available vaccines for COVID-19.

**Methods/design:**

We will conduct a living systematic review based on searches of major medical databases (e.g., MEDLINE, EMBASE, CENTRAL) and clinical trial registries from their inception onwards to identify relevant randomized clinical trials. We will update the literature search once a week to continuously assess if new evidence is available. Two review authors will independently extract data and conduct risk of bias assessments. We will include randomized clinical trials comparing any vaccine aiming to prevent COVID-19 (including but not limited to messenger RNA; DNA; non-replicating viral vector; replicating viral vector; inactivated virus; protein subunit; dendritic cell; other vaccines) with any comparator (placebo; “active placebo;” no intervention; standard care; an “active” intervention; another vaccine for COVID-19) for participants in all age groups.

Primary outcomes will be all-cause mortality; a diagnosis of COVID-19; and serious adverse events. Secondary outcomes will be quality of life and non-serious adverse events. The living systematic review will include aggregate data meta-analyses, trial sequential analyses, network meta-analyses, and individual patient data meta-analyses. Within-study bias will be assessed using Cochrane risk of bias tool. The Grading of Recommendations, Assessment, Development and Evaluations (GRADE) and Confidence in Network Meta-Analysis (CINeMA) approaches will be used to assess certainty of evidence. Observational studies describing harms identified during the search for trials will also be included and described and analyzed separately.

**Discussion:**

COVID-19 has become a pandemic with substantial mortality. A living systematic review assessing the beneficial and harmful effects of different vaccines is urgently needed. This living systematic review will regularly inform best practice in vaccine prevention and clinical research of this highly prevalent disease.

**Systematic review registration:**

PROSPERO CRD42020196492

**Supplementary Information:**

The online version contains supplementary material available at 10.1186/s13643-020-01516-1.

## Background

### Description of the condition

In 2019, a novel coronavirus named severe acute respiratory syndrome coronavirus 2 (SARS-CoV-2) caused a global outbreak of the respiratory illness called coronavirus disease 2019 (COVID-19) [1]. Since the initial outbreak in China, COVID-19 has been labeled the first pandemic to be caused by a coronavirus by the World Health Organization [2].

### Etiology

Coronaviruses are enveloped, positive-sense, and single-stranded RNA virus genomes [3–5]. The virus encodes a nonstructural replicase polyprotein and structural proteins, including spike (S), envelope (E), membrane (M), and nucleocapsid (N) [3–5]. The S protein on the surface of SARS-CoV is involved in receptor recognition and the attachment to and entry into human cells. It is therefore a common target for the development of vaccines and therapeutics [3, 5, 6].

Of the 30 coronaviruses that are known to infect mammals, birds, and other animals, seven are known to infect humans [4, 7]. Four of them usually causes mild diseases such as common cold (HKU1; OC43; 229E; and NL63), whereas Middle East respiratory syndrome (MERS)-CoV, SARS-CoV, and now SARS-CoV-2 are prone to cause more serious diseases [5, 7].

### Pathogenesis

SARS-CoV is mainly transmitted from person to person through respiratory droplets [8–10]. Its baseline reproduction number (the estimated number of people who will be infected by one contagious person) is estimated at 1.87 to 3.31 [9].

The clinical presentation of COVID-19 ranges from subclinical infection with mild, self-limiting respiratory tract illness to severe progressive pneumonia, multiorgan failure, and death [11–14]. Severe disease onset might result in death due to massive alveolar damage and progressive respiratory failure [12]. As of October 12, 2020, there were 37,423,660 confirmed patients, 1,074,817 confirmed deaths, and 216 countries, areas, or territories with COVID-19 according to the World Health Organization [15]. Some patients are suspected of having an increased risk of severe illness (e.g., people with chronic lung disease, serious heart disease, chronic kidney disease, elderly (above 65 years), and immunocompromised people) [16].

### How the vaccines might work

There is currently no vaccine for COVID-19 [17]. To control the growing COVID-19 pandemic, we currently rely on quarantine, isolation, and infection-control measures to prevent disease spread [18], and on supportive care including oxygen and mechanical ventilation for infected patients experiencing respiratory difficulty [19]. Today, the effects of numerous vaccines against severe acute respiratory syndrome coronavirus (SARS-COV-2) are being assessed in randomized clinical trials [17] (see Table 1).

**Table 1:** List of vaccines aiming to prevent COVID-19

Type of vaccine	Mechanism	Examples of ongoing trials (name of vaccine (study identifier))
RNA	Introduces RNA that codes for targets on the SARS-CoV-2 virus.	mRNA-1273 (NCT04283461, NCT04405076) BNT 162 (NCT04380701, NCT04368728)
Viral vector	Contains a viral (adenovirus) **vector** encoded with genetic information for the SARS-CoV-2 spike protein.	AZD 1222 (NCT04324606, NCT04400838)
DNA	This system introduces antigen-specific DNA into cells via plasmids to trigger T-cell and antibody response to the SARS-CoV-2 virus.	INO-4800 (NCT04336410) NVX-CoV2373 (NCT04368988)
Weakened/inactivated virus	This vaccine will use a weakened form of the virus that causes COVID-19.	PRO-nCOV-1001 (NCT04352608, NCT04352608)
Protein subunit	This recombinant 2019-nCoV S protein subunit-trimer vaccine relies on eliciting an immune response against the S-spike protein to prevent its docking with the host ACE2 receptor.	SCB-2019 (NCT04405908)
Dendritic cell vaccine	A vaccine consisting of autologous dendritic cells loaded with antigens from SARS-CoV-2, with or without GM-CSF	AV-COVID-19 (NCT04386252)
Viral proteins	This process will introduce viral proteins and immune modulatory genes to modify aAPCs and activate T-cell response.	aAPC (NCT04299724, NCT04276896)
BCG vaccine	BCG introduces weakened bacteria to trigger immune response, which may be effective against certain respiratory viruses.	BCG vaccine (NCT04328441, NCT04327206) VPM1002 (NCT04387409)

*SARS-CoV 2* Severe acute respiratory syndrome coronavirus 2, *RNA* ribonucleic acid, *DNA* deoxyribonucleic acid, *ASE2 receptor* Angiotensin-converting enzyme 2 receptor, *GM-CSF* Granulocyte macrophage-colony stimulating factor, *aAPCs* Artificial antigen presenting cells, *BCG* Bacillus Calmette-Guérin

Vaccines generally work by inducing the production of antibodies to prevent a microbial invasion [20, 21]. The antibodies achieve this by either neutralizing the pathogens or assisting the immune system with opsonization and/or phagocytosis capabilities [20, 21]. Alternatively, vaccines may focus on a cell-mediated or T-cell response to develop long-term immunity [22]. Animal studies suggest that vaccines inducing T-cell immune responses provide broad-spectrum immunity toward coronavirus infections [23, 24]. This may even make vaccines able to raise immunity toward future outbreaks of coronaviruses [23, 24].

The vaccines currently tested are based on different approaches to develop an immune response. The different vaccines use either mRNA [25, 26], DNA [27, 28], adenovirus vector [29–31], inactivated virus, weakened, or killed SARS-CoV-2 [32, 33], protein subunits [34], Bacillus Calmette-Guérin (BCG) vaccines, or other vaccines [35, 36]. Most vaccines attempt to train the immune system to recognize SARS-CoV-2’s S protein, which the virus uses to bind and enter host cells [3]. As described above, some vaccines focus on inducing T cell immunity [22–24].

### Why this review is important

The widespread COVID-19 paralysis of healthcare systems and societies worldwide is almost unprecedented. The pandemic has burdened most healthcare systems and has caused serious international economic challenges. There is currently no specific way of preventing the spread of the virus besides quarantine, isolation, and infection-control measures. There is therefore a need for an efficient vaccine to adequately prevent such pandemics now and in the future. WHO has stated that 70% efficacy of a future vaccine is preferred and 50% efficacy is considered a minimum requirement, assuming no serious adverse events [37].

A living systematic review of vaccines to prevent COVID-19 allows us to incorporate relevant new evidence as it becomes available, thereby decreasing the timespan from evidence to clinical practice, which is crucial in this international health crisis [38].

The development of an effective vaccine faces challenges as vaccine development takes about 10 years, and the typical success rate for upcoming vaccines is around 6% [17]. Some of the new techniques such as the nucleotide-based and adenovirus-based approaches have never produced a vaccine that has been approved in the USA or the EU [17]. A recent report concludes, in September 2020, that 321 vaccine candidates for COVID-19 exist globally [39]. Of these, 33 vaccine candidates are in clinical trials, with plans to enroll more than 280,000 participants from at least 470 sites in 34 different countries [39]. These include among others: Moderna’s mRNA COVID-19 vaccine [25]; CanSino’s non-replicating adenovirus type-5 (Ad5) vectored COVID-19 vaccine [40]; Beijing Institute of Biological Products’ Ad5-nCoV vaccine [30]; Inovio Pharmaceuticals’ DNA vaccine for COVID-19 [27]; an inactive COVID-19 vaccines manufactured by Sinovac [33]; University of Oxford’s non-replicating chimpanzee adenovirus vectored vaccine ChAdOx1 nCoV-19 [31]; and BioNTech’s mRNA COVID-19 vaccine [41].

We identified another important living review that is comparable to our present project [42]. It is a living mapping of ongoing randomized clinical trials with network meta-analysis on all interventions for COVID-19. This review includes both prevention, including vaccines, and treatments, but does not use trial sequential analysis or similar methods to handle problems with multiplicity (repeating updating of meta-analysis, multiple comparisons due to inclusion of multiple interventions, assessing multiple outcomes) [42, 43]. We have also identified living reviews that purely assess different therapeutic interventions for COVID-19 [19, 44].

The present living systematic review with aggregate data meta-analyses, trial sequential analyses, network meta-analyses, and individual patient data meta-analyses aims at forming the basis for evidence-based guideline recommendations for vaccines to prevent COVID-19, accounting for potential bias risks (systematic errors), random errors, and study design errors, as well as assessing certainty of our findings [43, 45–49].

## Methods

The protocol is reported in accordance with the reporting guideline provided in the Preferred Reporting Items for Systematic Reviews and Meta-Analysis Protocols (PRISMA-P) statement (see Additional file 1) [50, 51], and is registered in the International Prospective Register of Systematic Reviews (PROSPERO CRD42020196492) database. The review will be carried out following recommendations outlined in The Cochrane Handbook of Systematic Review of Interventions [46], and PRISMA [50, 52, 53].

### Criteria for considering studies for this review

#### Types of studies

We will search for and include randomized clinical trials, irrespective of publication status, publication year, and language. We will also include quasi-randomized studies and observational studies identified during our search for trials for the assessment of harms, but we will not conduct searches for these studies. The reason for including such observational studies is that randomized clinical trials often do not report rare adverse events or late-occurring adverse events.

#### Types of participants

Participants will be included irrespective of prior exposure, age, sex, comorbidities, immune status, and risk group.

#### Types of interventions

##### Experimental group

We will include any vaccine aiming to prevent COVID-19, i.e., all vaccines listed in Table 1 or any other vaccine irrespective of dose and duration of administration. We will group any vaccine with the same mechanism (e.g., RNA, DNA, viral vector, and protein subunit) and target (e.g., S-protein). Authors blinded to the data extraction of results will group the vaccines into groups with similar vaccines. The authors involved in this process will be blinded for authors of the trials and will not have access to the values of outcome data at this point.

##### Control group

We will include randomized clinical trials with any control group, i.e., head-to-head comparisons versus placebo, “active placebo” (a matching placebo that produces noticeable adverse effects that may convince the participant being vaccinated), usual care (or similar terms), no intervention, another vaccine aiming at preventing COVID-19, or any other “active” comparator. We will accept any of these control interventions irrespective of dose and duration of administration.

Co-interventions will be allowed provided they are administered equally to the comparison groups.

### Primary outcomes

1. All-cause mortality
2. Proportion of participants with confirmed COVID-19 (verified by RT-PCR or similar laboratory tests)
3. Proportion of participants with one or more serious adverse events. We will use the International Conference on Harmonization of technical requirements for registration of pharmaceuticals for human use—Good Clinical Practice (ICH-GCP) definition of a serious adverse event, which is any untoward medical occurrence that resulted in death, was life-threatening, required hospitalization or prolonging of existing hospitalization, and resulted in persistent or significant disability or jeopardized the participant [54]. If the trialists do not use the ICH-GCP definition, we will include the data if the trialists use the term “serious adverse event.” If the trialists do not use the ICH-GCP definition nor use the term serious adverse event, then we will also include the data if the event clearly fulfills the ICH-GCP definition for a serious adverse event. We will exploratorily assess each type of serious adverse event separately (see below).

### Secondary outcomes

1. Health-related quality of life (assessed on any valid continuous scale)
2. Proportion of participants with one or more adverse events not considered serious. We will exploratorily assess each type of adverse events not considered serious separately (see below)

### Exploratory outcomes

1. SARS-CoV-2 neutralizing antibody titers
2. SARS-CoV-2 IgG-binding antibody titers
3. Proportion of participants with seroconversion for SARS-CoV-2 neutralizing antibody (defined as either a fourfold increase from baseline or higher than 2 SDs above mean in the control group)
4. Proportion of participants with seroconversion for SARS-CoV-2 IgG-binding antibody (defined as either a fourfold increase from baseline or higher than 2 SDs above mean in the control group)
5. Individual types of serious adverse events will be analyzed separately
6. Individual types of adverse events not considered serious will be analyzed separately

We will use the trial results reported at maximum follow-up for all outcomes. We will also assess the time points 14 days and 28 days for the exploratory serological outcomes.

### Search methods for identification of studies

#### Electronic searches

An experienced information specialist will search Cochrane Central Register of Controlled Trials (CENTRAL), Medical Literature Analysis and Retrieval System Online (MEDLINE), Excerpta Medica database (EMBASE), Latin American and Caribbean Health Sciences Literature (LILACS), Science Citation Index Expanded (SCI-EXPANDED), Conference Proceedings Citation Index—Science (CPCI-S), Chinese Biomedical Literature Database (CBM), China Network Knowledge Information (CNKI), Chinese Science Journal Database (VIP), and Wafang Database to identify relevant trials. We will search all databases from their inception to the present. Trials will be included irrespective of language, publication status, publication year, and publication type. For a detailed search strategy for all electronic searches, see Additional file 2.

### Searching other resources

We will identify additional references by manually searching the references of articles from the computerized databases. We will also search special COVID-19 trial sites, including a website with living mapping and living systematic review of COVID-19 studies (https://covid-nma.com/), a website developed by Vaccine Centre at the London School of Hygiene & Tropical Medicine (https://vac-lshtm.shinyapps.io/ncov_vaccine_landscape/), The Lancet’s A real-time dashboard of clinical trials for COVID-19 (https://www.thelancet.com/journals/landig/article/PIIS2589-7500(20)30086-8/fulltext), the preprint server for health sciences, www.medrxiv.org, and an open, accessible, and frequently updated clinical trial registration for COVID-19 trials (10.12688/wellcomeopenres.15821.1).

We will also search online trial registries such as ClinicalTrials.gov (clinicaltrials.gov), the Chinese Clinical Trial Registry (www.chictr.org.cn), the European Medicines Agency (EMA) (www.ema.europa.eu/), the World Health Organization (WHO) International Clinical Trials Registry Platform (www.who.int/ictrp/), and the Food and Drug Administration (FDA) (www.fda.gov/) for ongoing or unpublished trials. We will contact experts in the field and pharmaceutical companies to enquire about additional trials. We will search for grey literature in the System for Information on Grey Literature in Europe OpenGrey (www.opengrey.eu).

### Data extraction and management

Two review authors will independently extract data from included trials in a predefined form. Disagreements will be resolved by discussion, or if required, through consultation with a third author (JCJ or CG). The two review authors will assess duplicate publications and companion papers of a trial together to evaluate all available data simultaneously (maximize data extraction, correct bias assessment). Each trial will be named after the first author and year of the primary publication and all secondary publications will be classified under that name. We will contact the trial authors by email to specify any missing data, which may not be reported sufficiently or not at all in the publication.

We will search for information regarding industry funding of either personal or academic activities for each trial author. We will note in the “Characteristics of included studies” table if outcome data were not reported in a usable way. Two review authors will independently transfer data into the Stata file [55].

### Living systematic review

A living systematic review is defined as a systematic review, which is continually updated and incorporates relevant new evidence as it becomes available [56]. This methodology may be particularly important in the COVID-19 pandemic, where research evidence is emerging rapidly, current evidence is uncertain, and new research may change policy or practice decisions [56].

**Fig. 1:**
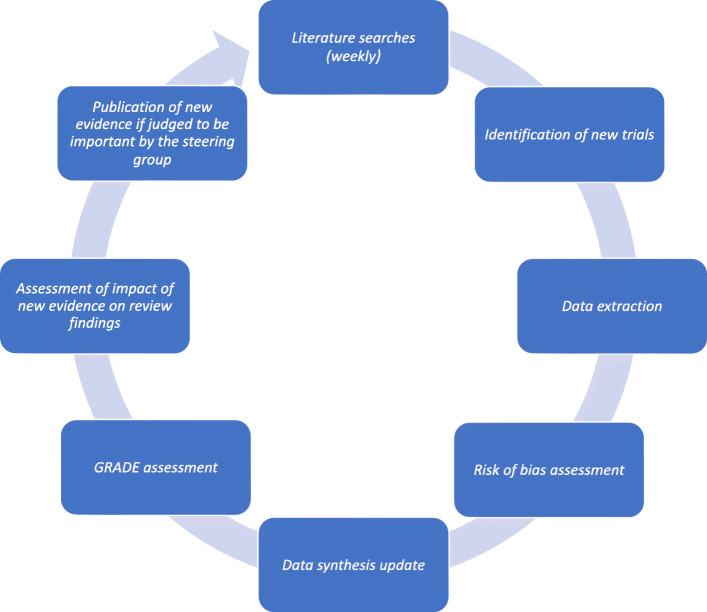
The living systematic review workflow

There are four fundamental differences between conventional systematic reviews and living systematic reviews: publication format, work processes, author team management, and statistical methods [57]. In this living systematic review, two independent investigators will receive an updated literature search file and include relevant newly published or unpublished trials once a week. The relevant meta-analyses, trial sequential analyses, and network meta-analyses will continuously be updated, and if new evidence is available (judged by the steering committee of the LIVING VACCINE review), the results will be published. Every month, the steering committee will discuss whether searching once a week is necessary. The living systematic review process will be initiated September 21, 2020. For an illustration of the living systematic review workflow, see Fig. 1 (with permission from Juul et al. [19] and Systematics Reviews).

### Trial characteristics

We will extract the following data: bias risk components (as defined below), trial design (parallel, factorial, crossover, cluster), estimation of sample size, inclusion and exclusion criteria, number of intervention groups, and length of follow-up.

### Participant characteristics

We will extract the following data: number of randomized participants, number of participants with comorbidities and types of comorbidities, number of analyzed participants, number of participants lost to follow-up/withdrawals/crossover, age range (mean or median), and sex ratio.

### Experimental intervention characteristics

We will extract the following data: type of vaccine, type of adjuvants, dose of intervention, and duration of intervention.

### Control intervention characteristics

We will extract the following data: type of control intervention, dose of intervention, and duration of intervention.

### Outcomes

All outcomes listed above will be extracted from each randomized clinical trial. For each outcome, we will identify if outcomes are missing, inappropriately measured, or selectively reported according to the criteria described later in the “missing outcome data” bias domain, the “risk of bias in measurement of the outcome” bias domain, and the “risk of bias in selection of the reported result” bias domain.

### Assessment of risk of bias in the included studies

Our bias risk assessment will be based on the Cochrane Risk of Bias tool—version 2 (RoB 2) as recommended in The Cochrane Handbook of Systematic Reviews of Interventions [46]. We will evaluate the methodology in respect of the following bias domains.

### Bias arising from the randomization process

#### Low risk of bias

Allocation was adequately concealed, AND there are no baseline imbalances across intervention groups at baseline appear to be compatible with chance, AND an adequate (random or otherwise unpredictable) method was used to generate allocation sequence, OR there is no information about the method used to generate the allocation sequence.

#### Some concerns

Allocation was adequately concealed, AND there is a problem with the method of sequence generation, OR baseline imbalances suggest a problem with the randomization process, OR no information is provided about concealment of allocation, AND baseline imbalances across intervention groups appear to be compatible with chance, OR no information to answer any of the signaling questions.

#### High risk of bias

Allocation sequence was not concealed, OR no information is provided about concealment of allocation sequence, AND baseline imbalances suggest a problem with the randomization process.

### Bias due to deviation from intended interventions

#### Low risk of bias

Participants, carers, and personnel were unaware of intervention groups during the trial, OR participants, carers, or personnel were aware of intervention groups during the trial but any deviations from intended intervention reflected usual practice, OR participants, carers, or personnel were aware of intervention groups during the trial but any deviations from the intended intervention were unlikely to impact on the outcome, AND no participants were analyzed in the wrong intervention groups (that is, on the basis of intervention actually received rather than of randomized allocation).

#### Some concerns

Participants, carers, or personnel were aware of intervention groups and there is no information on whether there were deviations from usual practice that were likely to impact on the outcome and were imbalanced between intervention groups, OR some participants were analyzed in the wrong intervention groups (on the basis of intervention actually received rather than of randomized allocation) but there was little potential for a substantial impact on the estimated effect of intervention.

#### High risk of bias

Participants, carers, or personnel were aware of intervention groups, and there were deviations from intended interventions that were unbalanced between the intervention groups and likely to have affected the outcome, OR some participants were analyzed in the wrong intervention groups (on the basis of intervention actually received rather than of randomized allocation), and there was potential for a substantial impact on the estimated effect of intervention.

### Bias due to missing outcome data

#### Low risk of bias

No missing data OR non-differential missing data (similar proportion of and similar reasons for missing data in compared groups) OR evidence of robustness of effect estimate to missing data (based on adequate statistical methods for handling missing data and sensitivity analysis).

#### Some concerns

An unclear degree of missing data or unclear information on proportion and reasons for missingness in compared groups AND there is no evidence that the effect estimate is robust to missing data.

#### High risk of bias

A high degree of missing data AND differential missing data (different proportion of or different reasons for missing data in compared groups) AND there is no evidence that the effect estimate is robust to missing data.

### Bias in measurement of outcomes

#### Low risk of bias

The outcome assessors were unaware of the intervention received by study participants, OR the outcome assessors were aware of the intervention received by study participants, but the assessment of the outcome was unlikely to be influenced by knowledge of the intervention received.

#### Some concerns

There is no information available to determine whether the assessment of the outcome is likely to be influenced by knowledge of the intervention received.

#### High risk of bias

The assessment of the outcome was likely to be influenced by knowledge of the intervention received by study participants.

### Bias arising from selective reporting of results

#### Low risk of bias

Reported outcome data are unlikely to have been selected, on the basis of the results, from multiple outcome measurements (e.g., scales, definitions, time points) within the outcome domain, and reported outcome data are unlikely to have been selected, on the basis of the results, from multiple analyses of the data.

#### Some concerns

There is insufficient information available to exclude the possibility that reported outcome data were selected, on the basis of the results, from multiple outcome measurements (e.g., scales, definitions, time points) within the outcome domain, or from multiple analyses of the data. Given that analysis intentions are often unavailable or not reported with sufficient detail, we anticipate that this will be the default judgment for most trials.

#### High risk of bias

Reported outcome data are likely to have been selected, on the basis of the results, from multiple outcome measurements (e.g., scales, definitions, time points) within the outcome domain, or from multiple analyses of the data (or both).

### Overall assessment of risk of bias

#### Low risk of bias

The trial is judged to be at low risk of bias for all domains for this result.

#### High risk of bias

The trial is judged to be at high risk of bias or to be at some concerns in at least one domain for this result. Our subgroup analysis will compare the intervention effect of trials at low risk of bias to trials at high risk of bias, that is one or more domains at some concern or high risk of bias.

We will assess the domains “missing outcome data,” “risk of bias in measurement of the outcome,” and “risk of bias in selection of the reported result” for each outcome result. Thus, we can assess the bias risk for each outcome assessed in addition to each trial. Our primary conclusions will be based on the results of our primary outcome results with an overall low risk of bias. Both our primary and secondary conclusions will be presented in the “Summary of findings” tables.

We will assess confidence in network meta-analysis results using CINeMA (Confidence in Net-work Meta-Analysis) [58–60].

### Differences between the protocol and the review

We will conduct the review according to this published protocol and report any deviations from it in the “Differences between the protocol and the review” section of the systematic review.

### Measurement of treatment effect

#### Dichotomous outcomes

We will calculate risk ratios (RRs) with 95% confidence interval (CI) for dichotomous outcomes, as well as the trial sequential analysis-adjusted CIs (see below). Peto’s odds ratio (OR) with 95% CI will be used where the number of observed events is small (less than 5% of sample per group), and treatment groups are balanced [61].

#### Continuous outcomes

We will calculate the mean differences (MDs) or in case of different measurement scales the standardized mean difference (SMD) with 95% CI for continuous outcomes. We will analyze change from baseline scores using a MD if the same scale is used across studies. For different measurement scales in the same analysis model, we will use the SMD effect size. In case some studies do not report change scores but provide follow-up values, we will combine them together in a single model using MD [46]. We will also calculate trial sequential analysis-adjusted CIs (see below).

### Dealing with missing data

We will use intention-to-treat data if provided by the trialists [62]. We will, as the first option, contact all trial authors to obtain any relevant missing data (i.e., for data extraction and for assessment of risk of bias, as specified above), when individual patient data is not available.

#### Dichotomous outcomes

We will not impute missing values for any outcomes in our primary analysis. In our sensitivity analyses (see the “Sensitivity analysis” section), we will impute data.

#### Continuous outcomes

If standard deviations (SDs) are not reported, we will calculate SDs using relevant trial data (e.g., *P* values), if available. We will prefer intention-to-treat data, but if the original report did not contain such data, per protocol data will be used. In our best-worst worst-best scenarios (see the “Sensitivity analysis” section) for continuous outcomes, we will impute data.

### Assessment of heterogeneity

We will primarily investigate forest plots to visually assess heterogeneity. We will secondly quantify heterogeneity using the *I*^2^ statistic [46, 63, 64] and will estimate the between-study variance using the restricted maximum likelihood method [65, 66]. We will investigate evident heterogeneity through subgroup analyses (see the “Subgroup analyses” section below). We may ultimately decide that a meta-analysis should be avoided if heterogeneity is high [46]. To assess the magnitude of heterogeneity, we will compare the estimated amount with the distribution by Rhodes et al. for continuous and Turner et al. for dichotomous data [67, 68].

### Assessment of reporting biases

We will use a funnel plot to assess reporting bias if ten or more trials are included [46]. We will visually inspect funnel plots to assess for small-study effects, accounting for its potential limitations (e.g., low power) [46]. From this information, we will assess possible reporting bias. For dichotomous outcomes, we will test asymmetry with the Harbord’s test [69] if *τ*^2^ is less than 0.1 and with the Rücker test if *τ*^2^ is more than 0.1 [46]. For continuous outcomes, we will use the regression asymmetry test [70] and the adjusted rank correlation [71].

### Unit of analysis issues

We will include randomized clinical trials for assessment of benefits and harms.

In case of trials with a cross-over design, we will include the data from the first trial period in order to avoid residual effects from the treatment [46]. In order to avoid repeated observations on trial participants, we will use participant trial data at the longest follow-up [46].

We will analyze cluster randomized trials using the procedures referenced in the *Cochrane Handbook for Systematic Reviews of Interventions* [46]. Where results did not control for clustering, we will contact trial authors to request an estimate of the intracluster correlation coefficient (ICC). If the trial authors are unable to provide an ICC, we will calculate the ICC using design effects [72].

If we during our searches for trials identify observational studies reporting on harms, we will tabulate these harms and report them separately in the “Results” section (in the review). This is to cover rare and late occurring harms. We will tabulate the types of adverse events (serious and non-serious) that are reported in the non-randomized studies retrieved with the searches for randomized clinical trials. This will limit the information on harms in our systematic review. If benefits of certain vaccines are found, then systematic reviews of harms, based on observational studies, should be conducted [73].

### Data synthesis

#### Aggregate data meta-analysis

We will undertake the aggregate meta-analyses according to The Cochrane Handbook of Systematic Reviews of Interventions [46], Keus et al. [74], and our eight-step assessment suggested by Jakobsen et al. [47]. We will use the statistical software Stata version 16.1 (command: meta) to analyze data [55]. We will assess our intervention effects with both a random-effects meta-analysis (DerSimonian and Laird method) [45] and fixed-effect meta-analysis (Mantel-Haenszel method) for each treatment comparison separately [75]. We will report the more conservative point estimate of the two [47]. The more conservative point estimate is the estimate with the highest *P* value or the widest confidence interval. If there is substantial deviation between the random-effects and fixed-effect meta-analyses, we will report and discuss the results. We will assess a total of three primary outcomes and two secondary outcomes, and we will therefore consider a *P* value of 0.0167 or less as the threshold for statistical significance [47]. We will investigate heterogeneity through subgroup analyses. We will use the eight-step procedure to assess if the thresholds for significance are crossed [47]. Where multiple trial arms are reported in a single trial, we will include only the relevant arms. If two comparisons are combined in the same meta-analysis, we will halve the control group to avoid double-counting [46]. Trials with a factorial design will be included. In case of, e.g., a 2 × 2 factorial designed trial, the two groups receiving COVID-19 vaccination will be considered experimental groups, while the two groups receiving a placebo, “active placebo,” standard care, no intervention, or “active” comparator will be considered control groups.

#### Trial sequential analysis

Due to the continuous inclusion of new trials and hence repetitive testing of accumulating data when updating reviews, there is an increased risk of type I error. We wish to control the risks of both type I errors and type II errors. We will therefore perform trial sequential analysis on all outcomes, in order to calculate the diversity-adjusted required information size (DARIS; that is, the number of participants needed in a meta-analysis to detect or reject a certain intervention effect) and the cumulative Z-curve’s breach of relevant trial sequential monitoring boundaries [48, 49, 76–82]. A more detailed description of trial sequential analysis can be found in the manual [76] and at http://www.ctu.dk/tsa/. For dichotomous outcomes, we will estimate the required information size based on the observed proportion of patients with an outcome in the control group (the cumulative proportion of patients with an event in the control groups relative to all patients in the control groups). When assessing the reduction of confirmed COVID-19, we will conduct three trial sequential analyses with a relative risk reduction of 20, 50, and 70% respectively. The 50% relative risk reduction will be our primary analysis. We will use a relative risk reduction of 20% for the remaining dichotomous outcomes, an alpha of 1.67% for all our outcomes, a beta of 10%, and the observed diversity as suggested by the trials in the meta-analysis. For continuous outcomes, we will in the trial sequential analysis use the observed standard deviation (SD), a mean difference equal to the observed SD/2, an alpha of 1.67% for all outcomes, a beta of 10%, and the observed diversity as suggested by the trials in the meta-analysis.

#### Network meta-analysis

We will obtain information about the interventions of interest either from head-to-head trials, or from trials comparing a COVID-19 vaccines with placebo, standard care, no intervention, or “active placebo.” Hence, the synthesis comparator set consists of all the vaccines listed in the background section as well as a placebo, “active placebo,” standard care, no intervention, or “active” comparator trials. Each specific vaccine will be analyzed separately and will also be clustered with similar vaccines. We will describe the characteristics of the eligible randomized clinical trials and their populations using frequencies and percentages for categorical data and means and standard deviations for continuous data.

Descriptive statistics will be also generated for each treatment comparison describing important clinical and methodological characteristics (e.g., publication year, participant age). Each outcome dataset will be presented in a different network diagram, where the size of the nodes will be proportional to the total number of randomized participants, and the width of each edge will be weighted according to the number of studies comparing the connected treatments. We will additionally plot the edges of each network according to the average risk of bias per treatment comparison, using green for low, yellow for moderate, and red for high risk of bias. We anticipate that any participant who meets inclusion criteria is, in principle, equally likely to be randomized to any of the interventions in the synthesis comparator set. Network meta-analysis will be performed using Stata 16.1 (command: mvmeta) under the frequentist framework [55] using the network suite of commands [83]. The network meta-analysis synthesizes evidence for the comparative effectiveness of more than two alternative interventions for the same condition [84]. In case we encounter trials with more than two arms included in our review, we will only include the study once in the table showing the “characteristics of included studies.” The latter will also prevent the problem of the trial appearing more than once in the risk of bias assessment and this way also ensures no double counting the number of randomized clinical trials.

We will only perform network meta-analysis if a connected network of trials can be conducted [85].

If network meta-analysis is possible, we will assess a priori the two prerequisite assumptions: transitivity and consistency. We will assess for the transitivity assumption across treatment comparisons in the network using boxplots and will evaluate the assumption of consistency using the design-by-treatment interaction model as a global test [63, 84]. Effect modifiers will be age, sex, ethnicity/origin, exposure to COVID-19, whether or not immunocompromised/ deficient, whether or not with chronic lung disease. The transitivity assumption for carrying out an NMA will be evaluated using these effect modifiers. We will also explore these through network subgroup meta-analyses (see the “Individual patient data meta-analysis” section below). If we conclude that the transitivity and consistency assumptions are not met, we will not perform network meta-analysis but will present direct and indirect evidence separately.

The estimation of each treatment comparison will be reported separately using the relevant effect size (RR), a 95% CI, and a 95% prediction interval. We will use the network forest plot to illustrate the summary effect size of the comparative effectiveness among interventions. Along the estimated effect sizes, we will present the ranking probabilities for each treatment being at each possible rank, as well as the surface under the cumulative ranking curve (SUCRA) [86, 87]. A rank-heat plot will be used to depict the SUCRA values (and their 95% CI) across all outcomes [88].

We will conduct a random-effects network meta-analysis, assuming a common within network heterogeneity for each analysis, since the nature of the interventions in the network is similar [83, 85].

With earlier iteration of the living review, it is possible that some of the networks may be very sparse, in which case between-study heterogeneity variances may be overestimated leading to wider credible intervals from network estimates [68, 89]. If wide CIs are due to rare events as well, we will use the Mantel Haenszel in NMA using R [90].

To make sure that this complex network meta-analysis will be meaningful, relevant, and manageable, we will use the following process to define the nodes of the network. We will independently and in duplicate extract all data from all trials. We will present all the extracted data minus the primary and secondary outcomes for another group of the authors, who based on lists of all the different vaccines (different types, adjuvants, doses, durations) and all the different comparators (different types, doses, durations) will determine the groupings of the meaningful nodes to be compared within each connected network. The authors involved in this process will be blinded for authors of the trials and will not have access to the values of outcome data at this point.

#### Individual patient data meta-analysis

Results of individual patient data meta-analysis will increase the possibility to identify subgroups of patients with specific effects of the assessed interventions [91–93]. It will enable us to calculate the treatment by covariate interactions using patient-level covariates (such as sex and age).

If we receive individual patient data for all eligible randomized clinical trials, we will analyze the data using a one-stage analysis model based on generalized linear mixed models. This analysis will be adjusted for the categoric baseline variables that the trials used as stratification variables in their randomization (only the common variables that all of the trials adjust for). When analyzing continuous data, we will also adjust all analyses for the baseline value.

If we are unable to obtain sufficient individual patient data, we will secondly conduct a two-stage analysis, where at 1st stage, we will reduce available individual patient data to aggregate data for each study, and at 2nd stage, we will combine all available data in a meta-analysis.

##### Assessments of underlying statistical assumptions

We will systematically assess underlying statistical assumptions for all statistical analyses [55, 94, 95]. In short, for all regression analyses, we will test for major interactions between each covariate and the intervention variable. We will, in turn, include each possible first-order interaction between included covariates and the intervention variable. For each combination, we will test if the interaction term is significant and assess the effect size. We will only consider that there is evidence of an interaction if the interaction is statistically significant after Bonferroni adjusted thresholds (0.05 divided by number of possible interactions) and if the interaction shows a clinically significant effect. If it is concluded that the interaction is significant, we will consider both presenting an analysis separately for each (e.g., for each site if there is significant interaction between the trial intervention and “site”) and an overall analysis including the interaction term in the model [55, 94, 95]. For a detailed description of the planned assessments for underlying assumptions, please consult the recommendations of Nørskov et al. [55, 94, 95].

### Subgroup analyses

We will perform the following subgroup analyses when analyzing the primary outcomes (all-cause mortality, confirmed COVID-19, and serious adverse events).

1. Trials at high risk of bias compared to trials at low risk of bias. This is due to literature demonstrating overestimation of benefits and underestimation of harms in trials at risk of bias [96–98].
2. Trials without for-profit bias compared to trials at unknown or known risk of for-profit bias. This is due to literature demonstrating overestimation of benefits and underestimation of harms in trials at risk of for-profit bias [99].
3. Type of vaccine (including but not limited to messenger RNA; DNA; non-replicating viral vector; replicating viral vector; inactivated virus; protein subunit; dendritic cell; other vaccines like live-attenuated; polysaccharide vaccine; conjugate vaccines).
4. Age (children and adolescents as defined by trialists; adults as defined by trialists; elderly as defined by trialists).
5. Type of antibody target (e.g., nonstructural replicase polyprotein, E protein, M protein, N protein, S protein, or other targets).
6. Ethnicity (e.g., Asian, Caucasian, Arab, Black, and Mixed) or participants’ origin (South East Asian, European, Eastern Mediterranean, African, Western Pacific).
7. Sex (male, female).
8. Trials including participants who are immunocompromised compared to trials with participants that are not.
9. Trials including participants with history of chronic lung disease compared to trials without history of chronic lung disease.
10. Trials including unexposed participants at the time of vaccination compared to trials including participants exposed to SARS-CoV-2.
11. Trials including vaccines that target a T cell-mediated response compared to trials that does not.
12. Trials using aluminum adjuvants compared to trials without aluminum adjuvants [100, 101].
13. Trials randomizing clusters compared to trials randomizing individual participants.
14. Trials using cross-over design compared to trials without cross-over design.

We will use the formal test for subgroup differences in STATA 16.1 (command: meta) [55]. We will perform any unanticipated subgroup analyses, if we identify these, as more information about this virus and its treatment becomes available. We will use ICEMAN to assess the credibility of the subgroups [102].

### Sensitivity analysis

To assess the potential impact of the missing data for dichotomous outcomes, we will perform the two following sensitivity analyses on all primary and secondary outcomes.

We will consider using multiple imputation techniques as recommended by Jakobsen et al. [62]. Please consult this publication for a detailed description of the handling of missing data. We will present best-worst and worst-best case scenarios if it is not valid to ignore missing data [47]. Best-worst and worst-best case scenarios assess the potential range of impact of the missing data for the trial results.

In the “best-worst” case scenario, it is assumed that all patients lost to follow-up in the intervention group have had a beneficial outcome, and all those with missing outcomes in the control group have had a harmful outcome [47]. Conversely, in the “worst-best” case scenario, it is assumed that all patients who were lost to follow-up in the experimental group have had a harmful outcome and that all those lost to follow-up in the control group have had a beneficial outcome [47]. When continuous outcomes are used, a “beneficial outcome” will be defined as the group mean plus two SDs of the group mean, and a “harmful outcome” will be defined as the group mean minus two SDs of the group mean [47].

We will present results of this scenario in our review. Other post hoc sensitivity analyses might be warranted if unexpected clinical or statistical heterogeneity is identified during the analysis of the review or any other unanticipated issues that we learn about COVID-19 along the way that may impact the results [47].

### Summary of findings tables

We will create summary of findings tables including each of the prespecified outcomes (all-cause mortality, confirmed COVID-19, serious adverse events, health-related quality of life, and non-serious adverse events). We will use the five GRADE considerations (bias risk of the trials, consistency of effect, imprecision, indirectness, and publication bias) and CINeMA to assess the quality of a body of evidence [47, 103–105]. We will assess imprecision using trial sequential analysis. We will downgrade imprecision in GRADE by two levels if the accrued number of participants is below 50% of the DARIS, and one level if between 50 and 100% of DARIS. We will not downgrade if the cumulative Z-curve crosses the monitoring boundaries for benefit, harm, or futility, or DARIS is reached. We will justify all decisions to downgrade the quality of evidence using footnotes, and we will make comments to aid the reader’s understanding of the review where necessary. Firstly, we will present our results in the summary of findings table based on the results from the trials with an overall low risk of bias, and secondly, we will present the results based on all trials. We will present the assessment of our three comparisons traditional aggregate data meta-analyses, network meta-analysis, and individual patient data meta-analyses separately. We will discuss all concurring results of these analyses as well as any conflicting results between the three. Two review authors will independently make judgments about the certainty of the evidence, with disagreements resolved by discussion or involving a third review author. We will justify, document, and incorporate judgments into reporting of results for each outcome. We will extract study data, format our comparisons in data tables, and prepare “Summary of findings” tables before writing the results and conclusions of our review.

### Data sharing and availability

Full syntax of all statistical analyses will be published as supplementary material. All aggregate data will be published regularly. Anonymized individual patient data will also be published if possible (we will discuss this with the trialists).

### Dissemination plan

Findings of this living systematic review will be published in international peer-reviewed scientific journals. Further, a dedicated webpage for the project will be developed, where iterative versions of the living systematic review will be accommodated with visual illustrations.

## Discussion

This living systematic review with aggregate data meta-analyses, trial sequential analyses, network meta-analyses, and individual patient data meta-analyses aims at comparing the effects of all vaccines for COVID-19 versus placebo, “active” placebo, standard care, no intervention, or an “active” intervention. Primary outcomes will be all-cause mortality, proportion of participants with confirmed COVID-19, and serious adverse events. Secondary outcomes will be health-related quality of life and proportion of participants with adverse events not considered serious.

This protocol has a number of strengths. The predefined methodology is based on the Cochrane Handbook for Systematic Reviews of Interventions [46], the PRISMA statements [50, 52, 53], the eight-step assessment suggested by Jakobsen et al. [47], trial sequential analysis [77], and GRADE assessments [104]. Hence, this protocol considers both risks of random errors and risks of systematic errors. Another strength of this protocol is that we plan to do a living systematic review, which allows us to continuously surveil the literature and update the evidence-base of existing vaccinations for preventing COVID-19 regularly resulting in a decreased timespan from evidence to clinical practice. This is particularly important in this international healthcare crisis. Furthermore, we plan to contact all trial authors to receive individual patient data. Often aggregate data meta-analyses and individual patient data meta-analyses tend to show similar overall results [93]. However, an advantage of us including individual patient data meta-analyses is that it may allow us to study intervention effects in subgroups of participants [92]. In addition, the synthesized evidence might be useful to evidence-based decision-making in healthcare. Thus, network meta-analyses should be activated to guarantee the quality of healthcare system.

Our protocol also has limitations. The primary limitation is the inclusion of all types of vaccines for the prevention of COVID-19. This may theoretically result in a large amount of comparisons resulting in problems with multiplicity. We plan to use trial sequential analysis to adjust thresholds for significance when continuously updating the review, but we do not take into account the large number of comparisons. This large risk of type 1 error will be considered when interpreting the review results.

The potential delay of negative and neutral results might also bias our results. Another limitation might be the publication of studies with questionable data that might lead to later retractions [106]. As a considerable proportion of ongoing trials are either conducted by pharmaceutical companies or have industry sponsorship, the results are at risk of “for-profit” bias [99]. That is industry-supported research is at risk of overstating benefits and understating harms [99, 107].

Our individual patient data meta-analysis might be limited to the availability of individual patient data.

Due to the large number of new trials, we might experience that the review could be outdated by the time it is publish, despite using the LIVING format.

Moreover, we primarily focus on randomized clinical trials and therefore primarily focus on benefits rather than harms as such trials are prone to miss rare and late occurring harms. When we identify observational studies reporting on harms during our searches for trials, we will include them separately in our results. We will tabulate the types of adverse events (serious and non-serious) that are reported in the non-randomized studies retrieved only with the searches for the randomized trials. However, this will limit the information on harms in our systematic review. If benefits of certain vaccines are found, then systematic reviews of harms, based on observational studies, should be conducted [73].

## Supplementary Information

**Additional file 1.** PRISMA-P 2015 Checklist

**Additional file 2.** Search strategies for Vaccines to prevent COVID-19: a protocol for a living systematic review with network meta-analysis including individual patient data (The LIVING VACCINE Project)

## Data Availability

Data sharing is not applicable to this protocol article. We will publish all data including code in the supplementary material of the systematic review.

## Abbreviations

BCG: Bacillus Calmette-Guérin
CBM: Chinese Biomedical Literature Database
CENTRAL: Cochrane Central Register of Controlled Trials
CI: Confidence interval
COVID-19: Coronavirus disease 2019
CPCI-S: Conference Proceedings Citation Index—Science
CNKI: China Network Knowledge Information
CSDR: Clinical Study Data Request
DARIS: Diversity-adjusted required information size
EMA: European Medicines Agency
EMBASE: Excerpta Medica database
FDA: US Food and Drug Administration
GRADE: The Grading of Recommendations Assessment, Development and Evaluation
ICH-GCP: International Conference on Harmonization of technical requirements for registration of pharmaceuticals for human use—Good Clinical Practice
MD: Mean difference
MEDLINE: Medical Literature Analysis and Retrieval System Online
MERS-CoV: Middle East respiratory syndrome
PRISMA: Preferred Reporting Items for Systematic Review and Meta-Analysis
PRISMA-P: Preferred Reporting Items for Systematic Review and Meta-Analysis – Protocols
PROSPERO: International Prospective Register of Systematic Reviews
RR: Risk ratio
RT-PCR: *Reverse transcription polymerase chain reaction*
SARS-CoV-2: Severe acute respiratory syndrome coronavirus 2
SCI-EXPANDED: Science index citation expanded
VIP: Chinese Science Journal Database
