# Supplementary material for: Vaccines to prevent COVID-19: a protocol for a living systematic review with network meta-analysis including individual patient data (The LIVING VACCINE Project)
Source: Syst Rev. 2020 Nov 20;9:262. doi: 10.1186/s13643-020-01516-1 (PMC7678579; doi:10.1186/s13643-020-01516-1)
Supplement: Supplementary file 2 — Additional file 2. Search strategies for Vaccines to prevent COVID-19: a protocol for a living systematic review with network meta-analysis including individual patient data (The LIVING VACCINE Project) [file 13643_2020_1516_MOESM2_ESM.pdf]

Search strategies for

Vaccines to prevent COVID-19: a protocol for a living systematic review with network meta-analysis including individual patient data (The LIVING VACCINE Project)

1. exp Coronavirus Infections/
2. ((corona adj (virus or viral or infection*)) or coronaviri* or covid* or sars-cov-2).mp. [mp=title, abstract, original title, name of substance word, subject heading word, floating sub-heading word, keyword heading word, organism supplementary concept word, protocol supplementary concept word, rare disease supplementary concept word, unique identifier, synonyms]
3. 1 or 2
4. exp Vaccines/
5. exp Vaccination/
6. exp Immunization/
7. (vaccin* or immun*).mp. [mp=title, abstract, original title, name of substance word, subject heading word, floating sub-heading word, keyword heading word, organism supplementary concept word, protocol supplementary concept word, rare disease supplementary concept word, unique identifier, synonyms]
8. ((RNA or DNA or recombinant* or vector* or inactivat* or live attenuat* or dendritic or adeno* or encapsulated or plasmid* or protein subunit or peptid* or spike) adj5 vaccin*).mp. [mp=title, abstract, original title, name of substance word, subject heading word, floating sub-heading word, keyword heading word, organism supplementary concept word, protocol supplementary concept word, rare disease supplementary concept word, unique identifier, synonyms]
9. (BNT162 or AZD1222 or Ad5* or INO-4800 or bacTRL* or PRO-nCov-100* or v-sars or vero cell* or SCB-2019 or NVX-COV2373 or BCG vaccin* or VPM1002 or aAPC or AV-COVID-19 or TBD* or Ad26* or MVA* or hAd5* or PiCoVacc or BBIBP-CorV).mp. [mp=title, abstract, original title, name of substance word, subject heading word, floating sub-heading word, keyword heading word, organism supplementary concept word, protocol supplementary concept word, rare disease supplementary concept word, unique identifier, synonyms]
10. 4 or 5 or 6 or 7 or 8
11. 3 and 10
12. (randomized controlled trial or controlled clinical trial).pt. or clinical trials as topic.sh. or trial.ti.
13. (random* or blind* or placebo* or meta-analys*).mp. [mp=title, abstract, original title, name of substance word, subject heading word, floating sub-heading word, keyword heading word, organism supplementary concept word, protocol supplementary concept word, rare disease supplementary concept word, unique identifier, synonyms]
14. 11 and (12 or 13)
